# Inflammatory Cytokines Associated with Diagnosis, Tumor Grade and Prognosis in Patients with Neuroendocrine Tumors

**DOI:** 10.3390/jcm11206191

**Published:** 2022-10-20

**Authors:** Lukas Geisler, Teresa Hellberg, Joeri Lambrecht, Henning Jann, Jana Knorr, Johannes Eschrich, Sven H. Loosen, Alexander Wree, Linda Hammerich, Andreas Krieg, Tom Luedde, Frank Tacke, Christoph Roderburg, Raphael Mohr

**Affiliations:** 1 Department of Hepatology and Gastroenterology, Campus Virchow Klinikum and Campus Charité Mitte, Charité Universitätsmedizin Berlin, 13353 Berlin, Germany; 2 Clinic for Gastroenterology, Hepatology and Infectious Diseases, University Hospital Düsseldorf, Medical Faculty of Heinrich Heine Universität Düsseldorf, 40225 Düsseldorf, Germany; 3 Department of Surgery (A), University Hospital Düsseldorf, Medical Faculty of Heinrich Heine Universität Düsseldorf, 40225 Düsseldorf, Germany

**Keywords:** neuroendocrine tumor, diagnosis, biomarker, cytokines, NETest

## Abstract

Background and aims: Inflammatory cytokines represent diagnostic and prognostic biomarkers in manifold cancers. Recent data suggest a pivotal role of these cytokines in different biological processes involved in the development of neuroendocrine tumors (NETs). However, their role as biomarkers in NETs is only poorly understood. Methods: We analyzed serum concentrations of 13 inflammation-related cytokines at different time points in 43 patients with well-differentiated gastroenteropancreatic NETs (G1/G2) treated at Charité Berlin and compared them to 40 healthy controls. The results were correlated with clinical records. Results: Serum concentrations (Median (Interquartile Range (IQR)) in pg/mL) of IL-1β (124 (82) vs. 68 (61) pg/mL; *p* = 0.0003), IL-6 (111(122) vs. 88 (32) pg/mL; *p* = 0.0086), IL-8 (1058 (768) vs. 210 (90) pg/mL; *p* < 0.0001), IL-18 (2936 (1723) vs. 1590 (704) pg/mL; *p* < 0.0001), and TNF (271 (260) vs. 42 (25) pg/mL; *p* < 0.0001) were significantly elevated in NET patients, whereas IL-10 (43 (44) vs. 105 (48) pg/mL; *p* < 0.0001) showed lower concentrations in NETs when compared to controls. Cytokine levels significantly correlated with tumor grade (IL-6; *p* = 0.0070), prevalence of distant metastasis (IL-18; *p* = 0.0313), and disease progression over time (IL-10; *p* = 0.0033) but not tumor location. Chromogranin A (CgA) and the NETest are currently used to monitor treatment response. A more accurate prediction could possibly be achieved by employing a subset of cytokines. Our data clearly warrants further functional investigation into the role of the immune response and cytokine release in NETs. Conclusion: A biologically plausible panel of cytokines might be added to the diagnostic and prognostic tools currently employed in patients with NETs. Combining different markers into a score would elevate diagnostic accuracy compared to single markers.

## 1. Introduction

Neuroendocrine tumors (NETs) represent a rare and heterogeneous group of tumors. The rising incidence throughout the last few decades may be mainly attributable to improvements in diagnostics, such as imaging and particularly nuclear medicine techniques [1,2]. The current incidence is estimated to be 0.3–0.6 cases per 100,000 for gastroenteropancreatic (GEP) NETs and 0.04–0.14 cases per 100,000 for GEP neuroendocrine carcinomas (NECs) [1,2].

GEP-NETs are derived from the diffuse endocrine system of the gastrointestinal tract and pancreas. Apart from their anatomical localization, they are categorized according to their histological differentiation and the Ki-67 proliferative index into low/moderate-grade (1/2 (G1/G2)) and high-grade (G3) NETs and NECs [3,4]. Well-differentiated NETs (G1/G2) typically have a low proliferative index and have an extraordinarily good prognosis compared to other malignancies [3,4]. Due to their asymptomatic behavior, GEP-NETs are frequently diagnosed in advanced stages, when curative surgical therapies are no longer available.

While multi-slice computed tomography (MSCT) was the diagnostic standard over a long period of time, magnetic resonance imaging (MRI) has recently gained importance. Moreover, advances in nuclear medicine using somatostatin-receptor-specific tracer imaging have improved diagnostic and therapeutic paradigms. Apart from all the progress in imaging techniques, extensive efforts have been undertaken to identify reliable serum-based diagnostic markers for GEP-NETs. At present, Chromogranin A (CgA) represents the most prominent marker in the context of NETs. However, it is more useful for the monitoring of tumor response to treatment, rather than for the initial diagnostic process. Molecular biomarkers, rather than secreted proteins, give an adequate picture of the biological activity of a tumor cell. In this context, the NETest, a standardized liquid biopsy evaluating the expression of 51 genes by real-time polymerase chain reaction (PCR), has proven its utility as a diagnostic tool. Nevertheless, its ability to predict prognosis and treatment response is discussed controversially [5]. The lack of easily accessible biomarkers is a major drawback in the early diagnosis of NETs. Therefore, innovative parameters reflecting novel pathophysiological concepts are eagerly needed to improve the clinical management of patients with NETs.

Cytokines are a class of small proteins (~5–25 kDa) with a pivotal role in the regulation of cell signaling. They are released in response to infection or inflammation and regulate tumor development and progression. Cancer cells respond to host-derived cytokines that can promote growth, attenuate apoptosis, and facilitate invasion and metastasis [6].

In neuroendocrine tumor cells, the signal transducer and activator of the transcription 3 (STAT3)/IL-6 axis has been implicated in proliferation, survival, and differentiation through MAPK-dependent signaling. [7,8]. Several studies suggesting its tumor-promoting role have been summarized in detail elsewhere [9]. Furthermore, a correlation between TNF, CgA, as well as NETs has already been suspected by Gregorc and colleagues [10]. It has been known to be increased in a multitude of malignancies, secreted by tumor-associated macrophages in the tumor microenvironment to promote disease progression by promoting epithelial-to-mesenchymal transition [11], but it may also induce apoptosis [12]. The caspase-1/IL-18 (IL-1ß) axis has additionally been evaluated in tumor-related disease. A recent study has shown that IL-18 correlates with the local Th1/tumor-infiltrating lymphocyte response in colorectal [13] as well as in breast cancer [14]. Moreover, it could be shown that the neutrophil-to-lymphocyte ratio and C-reactive protein [15,16], as well as other inflammatory cytokines, are regulated in the environment of neuroendocrine tumors [17]. Hence, we hypothesized that levels of pro-inflammatory cytokines, representing biologically plausible markers in the context of cancer, might be altered in patients with NETs. Therefore, we measured the serum levels of 13 cytokines in a well-characterized cohort of patients with NETs and correlated these results to the patients’ clinicopathological characteristics.

## 2. Materials and Methods

### 2.1. Design of Study and Patient Cohort

In this study, we longitudinally evaluated circulating levels of cytokines in a cohort of 43 patients with histologically proven NETs. NET patients who were admitted to the Charité, Universitätsmedizin Berlin, a tertiary healthcare center that provides specialized care to patients with NETs, were prospectively recruited between 2011 and 2017. Patients were consecutively included during routine care visits, and no further inclusion/exclusion criteria beyond the diagnosis of NET were applied. Serum was collected at four different time points over one year and retrieved using centrifugation at 2000 rpm/10 min at room temperature. In this paper, we only used data obtained at baseline measurements (BL) at study inclusion and follow-up measurements (FU) after 12 months. To avoid repeated freeze–thawing, serum aliquots were snap-frozen at −80 °C until further use. In this study, 40 healthy blood donors, who showed no evidence of a malignant tumor, served as control samples. Patients were included into the study upon providing written informed consent. The study protocol was approved by the Ethics committee of Charité Berlin (ethical approval number EA1/229/17).

### 2.2. Imaging

Staging and evaluation of tumor burden was conducted by computed tomography, magnetic resonance imaging, DOTATOC-PET/CT, and in some cases by endosonography. The best procedure in each individual case was determined by the tumor conference in an interdisciplinary consensus. For the diagnosis of metastatic disease, mainly somatostatin-receptor-based imaging was used. Treatment response was assessed using the RECIST guidelines.

### 2.3. Multiplex Evaluation of Serum Cytokines

Evaluation of cytokine content in serum samples was performed using the LEGENDplex Human Inflammation Panel 1 (Biolegend, San Diego, CA, USA) according to the manufacturer’s protocol using technical duplicates. Measurements were performed using a BD FACSCanto™ II (BD Biosciences, Franklin Lakes, NJ, USA) with standard settings in the APC and PE channels. The LEGENDplex Human Inflammation Panel 1 includes the following cytokines: IL-1β, IFN-α2, INF-γ, TNF-α, CCL2 (MCP-1), IL-6, CXCL8 (IL-8), IL-10, IL-12p70, IL-17A, IL-18, IL-23, and IL-33. Concentrations were given as (Median [Interquartile Range (IQR)] in pg/mL).

### 2.4. Measurement of Standard of Care Biomarkers

Chromogranin A measurements were performed via fluoroimmunoassay using time-resolved amplified cryptate emission (TRACE) according to the standard procedures of our local central laboratory. Of note, in all cases where this was medically justifiable, PPI therapy was interrupted at least 14 days before a CgA measurement to avoid falsification of the measurement. The NETest comprises a two-step protocol with RNA isolation, complementary deoxyribonucleic acid (cDNA) production, and quantitative PCR from EDTA-collected whole blood. Target transcript levels are normalized and quantified versus a population control [18].

### 2.5. Statistics

Statistical calculations were performed using GraphPad Prism (version 9.3.1; GraphPad, La Jolla, CA, USA) and MedCalc version 18 (MedCalc Software, Ostend, Belgium). Normality was evaluated using the Shapiro–Wilk test as well as the Kolmogorov–Smirnov test. The Mann–Whitney test was performed to compare non-parametric data between two groups, whilst the Kruskal–Wallis with Dunn’s multiple comparison test was employed to evaluate the variance between multiple groups. A *p*-value < 0.05 was considered statistically significant (* *p* < 0.05; ** *p* < 0.01; *** *p* < 0.001; **** *p* < 0.0001). AUC was automatically calculated using GraphPad Prism’s implemented ROC analysis/calculation function with standard settings (Clopper–Pearson method, 95% confidence interval, ROC curve reported in percent). Diagnostic algorithms were created based on logistic regression analysis, using MedCalc version 18.

## 3. Results

### 3.1. Patient Characteristics

We included 43 patients with histopathologically confirmed NETs. The median age was 68.8 years, with 22 females and 21 males (Table 1). In this study, 47% of patients had a pancreatic primary tumor, whilst the remaining tumors originated from the ileum. It was found that 33% of all tumors were G1, whilst 67% were G2 tumors. No patients with NEC were included. TMN stage IV was most prevalent (72%), and a majority (84%) of patients were found to have distant metastasis. At the time of first serum sampling, 79% of the patients with G1 tumors were receiving somatostatin analogue (SSA) therapy. Of the G2 NET patients, 28% were treated with SSA, 24% with everolimus, and 10% received cytostatic chemotherapy with temozolomide plus capecitabine (TemCap). Only 34% of all patients with G2 NET underwent surgery, and subsequently, systemic antitumoral therapy was initiated. During the one-year period of observation, half of our patients (53%) experienced a tumor progression.

### 3.2. Serum Levels of IL-1β, IL-6, IL-8, IL-10, IL-18, and TNF Are Altered in Patients with Neuroendocrine Tumors

**Figure 1:**
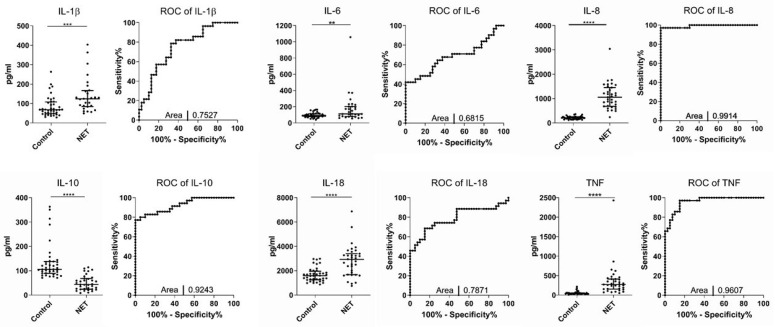
Inflammatory serum cytokines between healthy controls and NET patient cohort. Analysis of serum cytokines in healthy controls and patients with pancreatic or ileal NETs. Receiver-operating characteristic (ROC)/area under the curve was used to indicate that all evaluated cytokines allow for a discrimination between NET patients and healthy controls (** *p* < 0.01, *** *p* < 0.001, **** *p* < 0.0001). Horizontal bars indicate median and IQR; AUC was automatically calculated using GraphPad Prism.

We first compared serum levels of different inflammatory cytokines between healthy controls and patients with NETs. Notably, these analyses revealed significantly higher levels of IL-1β (124 (82) vs. 68 (61) pg/mL; *p* = 0.0003), IL-6 (111 (122) vs. 88 (32) pg/mL; *p* = 0.0086), IL-8 (1058 (768) vs. 210 (90) pg/mL; *p* < 0.0001), IL-18 (2936 (1723) vs. 1590 (704) pg/mL; *p* < 0.0001), and TNF (271 (260) vs. 42 (25) pg/mL; *p* < 0.0001), whereas concentrations of IL-10 (43 (44) vs. 105 (48) pg/mL; *p* < 0.0001) were lower in NETs when compared to controls (Figure 1).

Of note, due to the small set of patients, we were not able to age- or sex-match our control and study cohorts. When comparing the results between genders in the study and control cohort, no significant differences were shown. As expected, baseline cytokine levels were slightly higher in males, while this trend was reversed in the NET cohort for IL-18 and TNF only (Supplementary Figure S1). Consecutively, we performed ROC curve analyses, showing that IL-8 (AUC = 0.99), IL-10 (AUC = 0.92), and TNF (AUC = 0.96) represent strong indicators for the inflammatory phenotype present in NETs, while the diagnostic value of IL-1β (AUC = 0.75), IL-6 (AUC = 0.68), and IL-18 (AUC = 0.78) between the control cohort and NET cohort was less apparent (Figure 1). Combining the circulating levels of IL-8, IL-10, and TNF into a logistic regression algorithm created a high diagnostic value for discriminating between control and NET patients (AUC = 1.00).

Diagnostic Score=(0.098831 ∗ TNF)+(0.22409 ∗ IL−8)−(0.067070 ∗ IL−10)−95.65043 TNF, IL-8, and IL-10 in pg/mL

### 3.3. NETest and IL-6 Levels Can Discriminate between G1 and G2 NET

**Figure 2:**
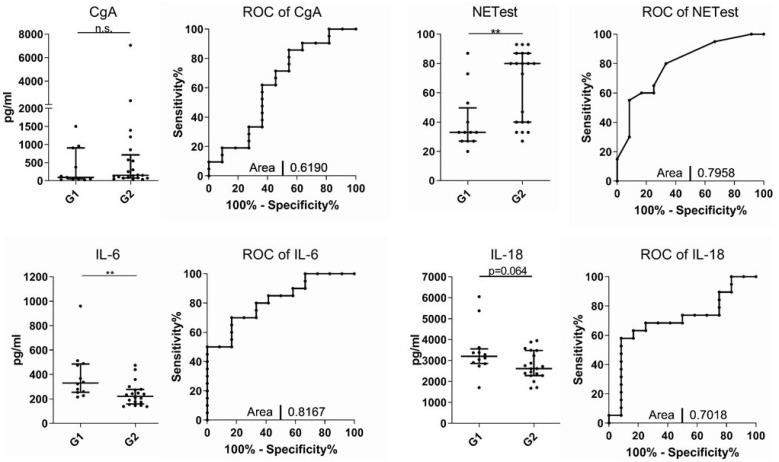
IL-6 serum cytokine levels indicate a difference between Grade 1 and 2 tumors. Analysis of serum cytokines between patients with G1/G2 NETs at the beginning of the study (baseline; BL). Receiver-operating characteristic (ROC)/area under the curve to indicate that all evaluated cytokines allow a discrimination between G1 and G2 tumors (** *p* < 0.01). Horizontal bars indicate median and IQR; AUC was automatically calculated using GraphPad Prism.

We hypothesized that concentrations of both established NET biomarkers and our novel subset of cytokines might reflect basic tumor characteristics such as tumor grading. Dividing the cohort according to the histopathological grading into G1 and G2 tumors, we compared levels of CgA and the NETest as well as concentrations of IL-1β, IL-6, IL-8, IL-10, IL-18, and TNF. Notably, CgA did not discriminate between grade 1 and 2 tumors, while the NETest was significantly higher in patients with G2 tumors than in patients with G1 tumors (33 (23) vs. 80 (47); *p* = 0.0043; Figure 2). Of all the analyzed cytokines, only IL-6 concentrations varied significantly with respect to the tumor grading since patients with G2 tumors displayed significantly lower IL-6 levels compared to patients with G1 NETs (221 (119) vs. 330 (231) pg/mL; *p* = 0.0070; Figure 2). IL-18 showed a similar pattern but failed to reach statistical significance in G2 vs. G1 tumors (G2: 2614 (1200) vs. G1: 3203 (700) pg/mL; *p* = 0.0644; Figure 2).

### 3.4. Tumor Localization Does Not Influence Inflammatory Cytokine Levels

We subsequently analyzed the influence of the anatomical tumor localization on concentrations of CgA, the NETest, as well as IL-1β, IL-6, IL-8, IL-10, IL-18, and TNF (Supplementary Figure S2). In these analyses, no significant differences between ileal and pancreatic primary tumors were apparent. Finally, we analyzed whether these markers might reflect the tumor stage according to the presence of distant metastases or not. Only IL-18 concentrations were altered in the distant metastases group with higher levels indicating the presence of distant metastatic diseases (3372 (1189) vs. 2799 (527) in pg/mL; *p* = 0.0313; Supplementary Figure S3).

### 3.5. Inflammatory Cytokines Correlate with the Course of the Disease

**Figure 3:**
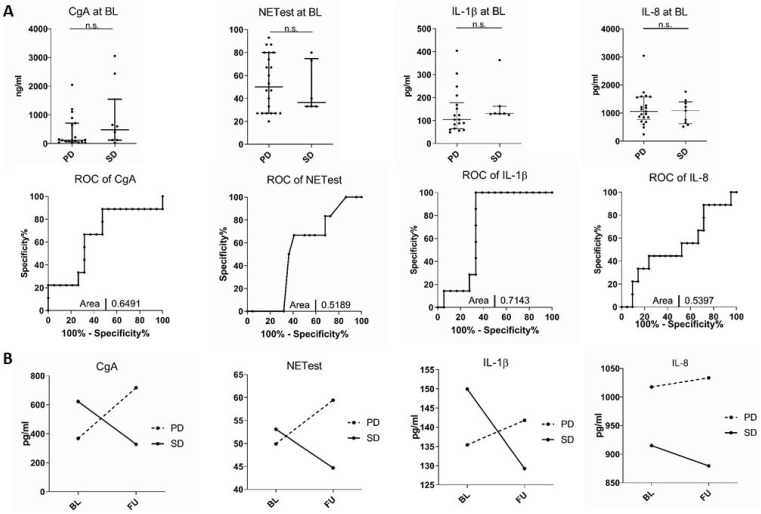
Serum cytokine levels at initial diagnosis may indicate the course of disease. (**A**) Analysis and ROC of serum cytokines between NET patients at the initial time point between progressive disease (PD) and stable disease (SD). (**B**) Time-course cytokine data sorted by disease status at the baseline (BL), initial follow-up (FU), and follow-up (FU) that was observed in the preparation of this study. Shown are the average values of patients with stable/remissive disease (full) and progressive (dotted line) disease. Horizontal bars indicate median and IQR; AUC was automatically calculated using GraphPad Prism.

We aimed to evaluate whether serum levels of IL-1β, IL-6, IL-8, IL-10, IL-18, and TNF, as well as CgA or the NETest, might serve as prognostic biomarkers in patients with NETs. We therefore compared serum concentrations of all markers at baseline before the initiation of therapy between patients that had stable disease versus patients with disease progression before the initial drawing of blood. Therapy itself had no significant impact on serum cytokines (Supplementary Figure S4). Neither inflammatory cytokine levels nor CgA or NETest values at baseline showed significant differences when compared with disease progression (Figure 3A). The findings were consistent independent of treatment groups, including surgical cases (Supplementary Figure S4). Thus, single biomarker values at a sole point in time seem insufficient for prognostic purposes of the course of the disease.

On the other hand, values of CgA and NETest increased over the time of observation in patients with progressive disease and decreased in patients with stable disease (Figure 3B) without statistical significance. Notably, a similar pattern was observed for IL-1β and IL-8, while the other cytokines did not correlate with the course of disease (Figure 3B).

**Figure 4:**
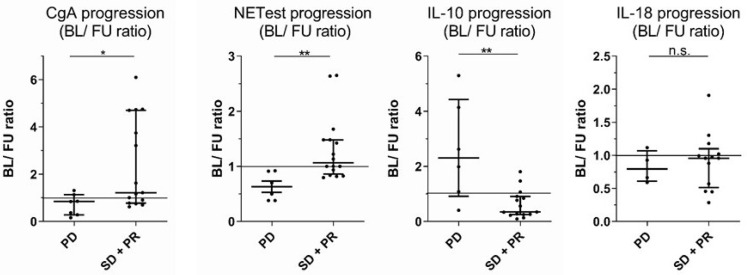
Cytokine ratios may hint at disease progression. Evaluation of disease progression according to the analyte ratio between initial the baseline (BL) and follow-up (FU) for each patient. Shown are patients with PD or the combined group of SD and partial remission (PR) (* *p* < 0.05, ** *p* < 0.01). Horizontal bars indicate median and IQR; AUC was automatically calculated using GraphPad Prism.

We calculated ratios between baseline and follow-up (FU) analyses. In these analyses, ratios were significantly different for NETest (PD: 0.6 (0.53) vs. SD + PR: 1.07 (0.62); *p* = 0.0016) and IL-10 (2.31 (3.51) vs. 0.35 (0.66); *p* = 0.0033; Figure 4). Therefore, we assumed that IL-10 may be a discriminating factor for disease progression, which is in line with the findings of Alvarez and colleagues, who identified IL-10 as an essential regulator for NETs [19].

## 4. Discussion

By analyzing a well-characterized cohort of patients with well-differentiated NETs, we demonstrate that a biologically plausible set of cytokines consisting of IL-1β, IL-6, IL-8, IL-10, IL-18, and TNF might be useful in the diagnostic process of neuroendocrine malignancies. IL-6 levels might correlate with the histopathological grading of NETs. Serum concentrations of inflammatory cytokines at a single point in time do not correlate with the clinical course of the disease. Nevertheless, a longitudinal measurement of IL-1β and IL-8 might serve as biomarkers to monitor tumor activity.

The initial diagnosis and evaluation of GEP-NETs remain a major challenge. While standard care includes different types of imaging, new strategies including more reliable and more easily accessible biomarkers are a key focus of ongoing research. Those methods are often termed liquid biopsy, as they allow for a closer evaluation of disease progression in blood or serum samples. Chromogranin A is still used as reference marker for GEP-NET. Nevertheless, no singular marker has yet been proven to be reliable for the diagnosis or prognosis of the disease. As an example, CgA is not necessarily secreted in all NETs, limiting its value for disease management [20,21]. Current studies have investigated the effect of microRNA (miRNA) signatures, which may prove helpful when used in multianalyte approaches [22,23]. PCR-based evaluation of NET transcriptomics was initially described in 2014. This involved the analysis of 51 targets in serum samples providing a better sensitivity (>95%) and specificity compared to CgA. Other approaches are evaluating novel targets [24], while much of the attention is focused on evaluating the immune system to determine predictive scores [25,26] or the regulatory activity to identify master regulators whose activity may be altered during targeted immunotherapy [19]. Our retrospective study evaluated the development of serum cytokines, especially those relevant to inflammatory processes in patients with diagnosed GEP-NET. It is apparent that all analytes, except the anti-inflammatory IL-10, were upregulated in comparison to healthy controls, most likely due to a complex interaction between the stromal tumor microenvironment and inflammatory infiltrates, which has already been described for other tumor identities [27,28] as well as for GEP-NET [29]. Especially, the prognostic role of the neutrophil-to-lymphocyte ratio has been discussed in more detail [30]. IL-8, which had the highest AUC value ROC in our study compared to healthy controls was also discussed in detail [31] and is known as a neutrophil chemoattractant in the tumor microenvironment in a multitude of human cancers [32]. Especially for NETs, it is known to be upregulated in the pancreatic tumor microenvironment [33].

Thus, our data are in line with those in previous reports. By performing subgroup analyses, we neither found significant differences between ileal and pancreatic NETs nor differences according to treatment groups. Nevertheless, we found a significant upregulation of IL-18 in patients with metastatic disease. A similar trend without statistical significance was visible in the NETest (*p* = 0.34), CgA, and IL-1β. The pro-tumorigenic role of IL-18 has been well-studied [34,35,36], and its high availability in the serum makes it a prime candidate as an indicator for epithelial reaction and metastasis formation [37,38,39]. Previous research has already established the role of IL-18 in mediating the Th1/tumor-infiltrating lymphocyte response [13]. Similarly, high levels of IL-6 in hepatocellular carcinoma correlate with poor survival prognosis and recurrence [40]. Interactions influencing the neuroendocrine differentiation markers NSE and CgA through differentiation RE1-silencing transcription factor have been discussed previously [41]. In contrast, we found a significant reduction of IL-6 and less pronounced reduction of IL-1β, IL-8, and IL-18 in patients with grade 2 tumors. Only TNF and CgA followed the trend of the NETest, which showed significant differences between tumor grades.

In the following, we started looking into the potential of serum markers for predicting disease status and disease progression. Quite surprisingly, NETest and CgA values did not significantly differentiate between stable and progressive disease in our study, while longitudinal observations show that they are strongly positively associated with disease status. The same holds true for IL-1β, where we found a marked decrease over time in patients with stable disease compared to an increase in patients with PD. Calculating BL/FU ratios showed a marked difference between PD and SD + PR groups for IL-10. The anti-inflammatory role, limiting the host’s immune response and thereby promoting tumorigenesis, has been widely established [42], e.g., limiting the Th1 immune response [43]. Still, IL-10 has been shown to directly affect the expression of neuroendocrine markers in experimental research, hinting towards an additional role in NETs and warranting additional research [44].

While we believe our study may show the potential of evaluating serum cytokines in NETs, we know it has limitations due to several reasons. It was performed retrospectively and is single-centered, with a relatively small cohort of patients. Hence, we acknowledge that further and more detailed workups in multi-centered studies would be required to delineate the importance of inflammatory cytokines in NET disease progression on a larger scale. As NETs are rare diseases with heterogeneous phenotypes depending on tumor origin, finding a coherent, reliable (immunological) biomarker for all subgroups appeared difficult. This is why we chose to limit the study cohort to G1/G2 pancreatic/ileal NETs, while it would have been of major interest to contrast that patient data with well-differentiated G3 tumor-bearing patients. In addition, our study solely included the evaluation of serum parameters aside from the routine clinical workup. While these are easily accessible, additional histological evaluation to identify possible differences or sources of inflammatory cytokines would likely be helpful to identify inter-patient variables. This is of specific importance since serum markers (e.g., CgA) might be biased by patient-specific factors such as the patient’s medication (e.g., PPI).

We do not want to argue against applying established scores or biomarkers for disease diagnosis and monitoring. Nevertheless, we believe that the implementation of inflammatory cytokines as biomarkers could have an important clinical benefit. Immune regulation has a pivotal role in the NET microenvironment. Nevertheless, the specific players and interactions seem poorly understood. Further mechanistical investigation is urgently needed in order to improve diagnostic and therapeutic options for patients with NETs.

## 5. Conclusions

In summary, our data show that inflammatory cytokines may be used to evaluate disease characteristics and indicate disease status. A scoring system including such values, in combination with the NETest, may be useful to evaluate disease progression more easily and cost-effectively.

## Figures and Tables

**Table 1:** Patient and control characteristics. If not indicated otherwise, data are shown as *n* (%) of patients or median and range. Percentages were rounded and may not sum to 100%. Table includes (**A**) patients with NETs of gastroenteropancreatic origin and (**B**) healthy controls.

NET Cohort (A)		Control Cohort (B)	
Age at initial sample (in years) Median/Range Female (Median/Range) Male (Median/Range)	68.8/42.1–87.9 71.9/60.7–80.5 67/42.1–87.9	Age at initial sample (in years) Median (Range) Female (Median/Range) Male (Median/Range)	40/23–77 46/23–77 38/24–68
Sex female male	22 (51.2%) 21 (48.8%)	Sex female male	10 (33.3%) 30 (66.6%)
Tumor location Illeum Pancreas	23 (53.5%) 20 (46.5%)		
Grading G1 G2	14 (32.6%) 29 (67.4%)		
TNM stage II III IIIB IV	1 (2.3%) 9 (20.9%) 2 (4.7%) 31 (72.1%)		
Metastasis Lymph node only Distant metastasis	9 (16%) 34 (84%)		
Treatment at initial sample G1 therapy SSA OP G2 therapy SSA OP PRRT Everolimus Tem/Cap	14 (100%) 11 (78.5%) 3 (21.4%) 29 (100%) 8 (27.6 %) 10 (34.4%) 1 (3.4%) 7 (24.1%) 3 (10.3)		

## Data Availability

The data presented in this study are available on reasonable request from the corresponding author. Due to sensitivity of human data derived of long-term patients of Charité Berlin the data are not made publicly available.

## Supplementary Materials

The following supporting information can be downloaded at: https://www.mdpi.com/article/10.3390/jcm11206191/s1. Supplementary Figure S1: Evaluation of serum cytokines at BL in NET according to sex. Supplementary Figure S2: Evaluation of serum cytokines at BL according to the prevalence of metastasis. Supplementary Figure S3: Evaluation of serum cytokines at BL according to the prevalence of metastasis. Supplementary Figure S4: Evaluation of serum cytokines at FU in NET according therapy option.

## Abbreviations

AUC	Area under the curve
CgA	Chromogranin A
cDNA	Complementary deoxyribonucleic acid
GEP	Gastroenteropancreatic
IL	Interleukin
INF	Interferon
IQR	Interquartile range
kDa	Kilodalton
MCP	Macrophage chemoattractant protein
mRNA	Messenger RNA
miRNA	MicroRNA
MRI	Magnetic resonance imaging
MSCT	Multi-slice computed tomography
NEC	Neuroendocrine carcinoma
NET	Neuroendocrine tumor
PCR	Polymerase chain reaction
PD	Progressive disease
ROC	Receiver-operating characteristic
SD	Stable disease
SSR	Somatostatin receptor
TemCap	Temozolomide plus Capecitabine
Th1	T helper type 1
TNF	Tumor necrosis factor
TRACE	Time-resolved amplified cryptate emission
