# Supplementary material for: Inflammatory Cytokines Associated with Diagnosis, Tumor Grade and Prognosis in Patients with Neuroendocrine Tumors
Source: J Clin Med. 2022 Oct 20;11(20):6191. doi: 10.3390/jcm11206191 (PMC9604855; doi:10.3390/jcm11206191)
Supplement: Supplementary file 1 [file jcm-11-06191-s001.zip › jcm-1867510-supplementary.pdf]

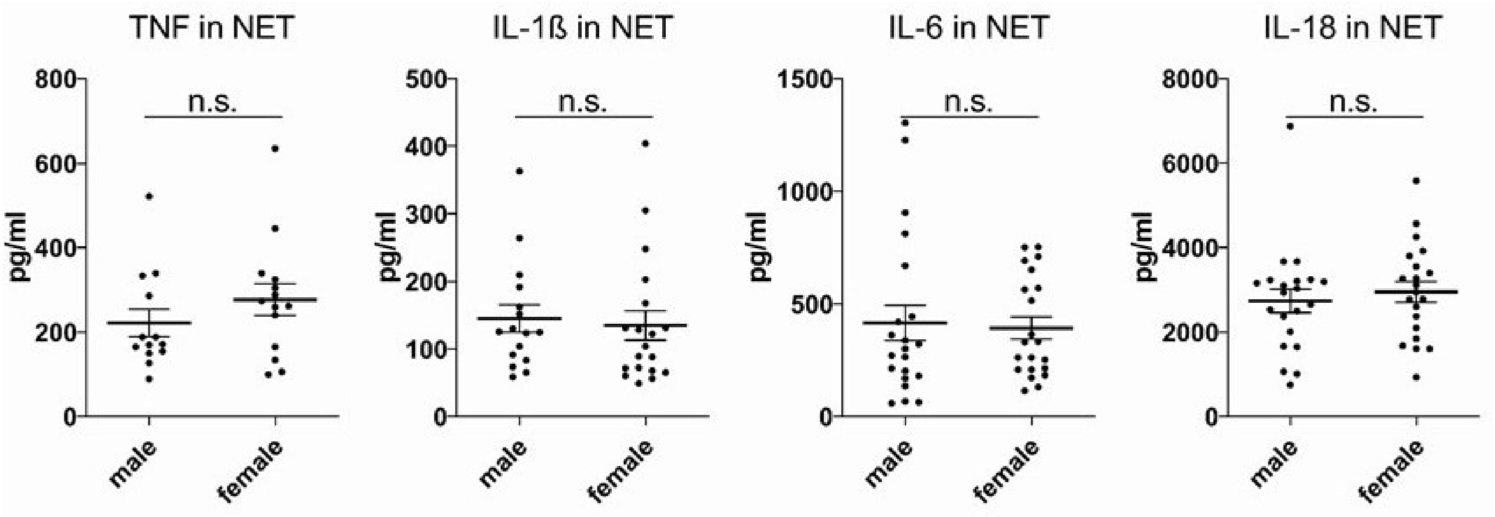

Supplementary Figure S1. **Evaluation of serum cytokines at BL in NET according to sex.** (* p <0.05), horizontal bars indicate median and IQR)

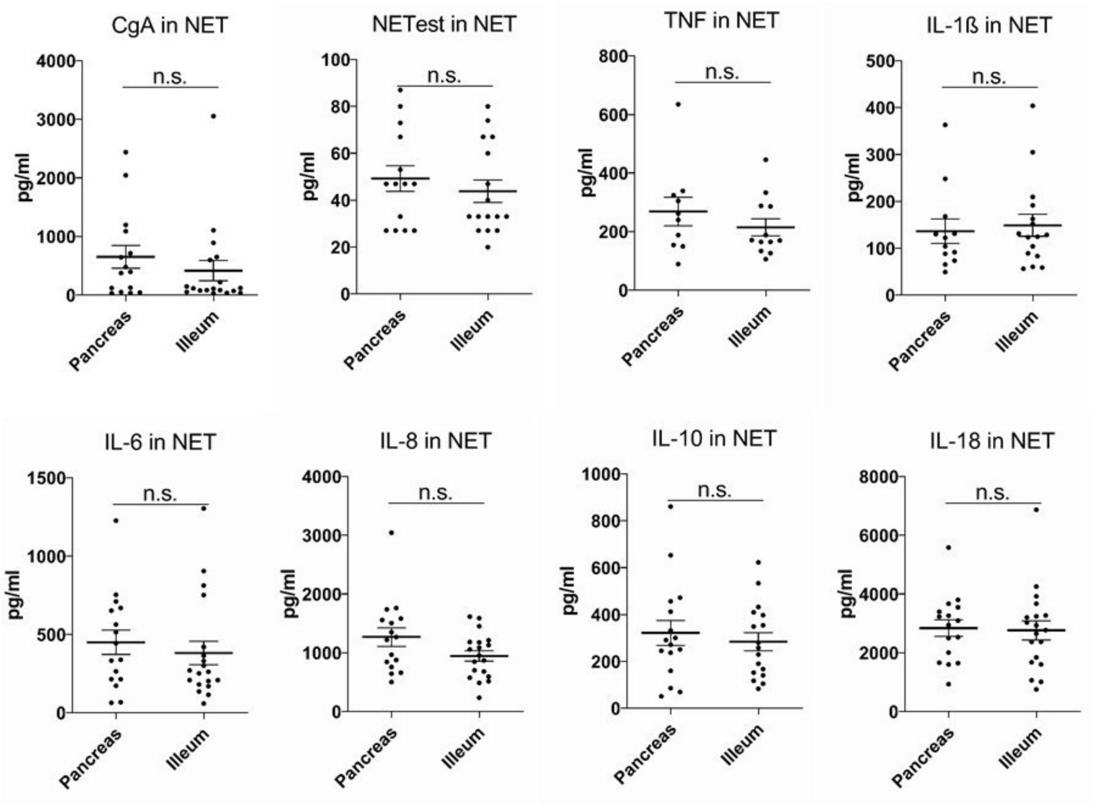

Supplementary Figure S2. **Evaluation of serum cytokines at BL according to the prevalence of metastasis.** (* p <0.05), horizontal bars indicate median and IQR)

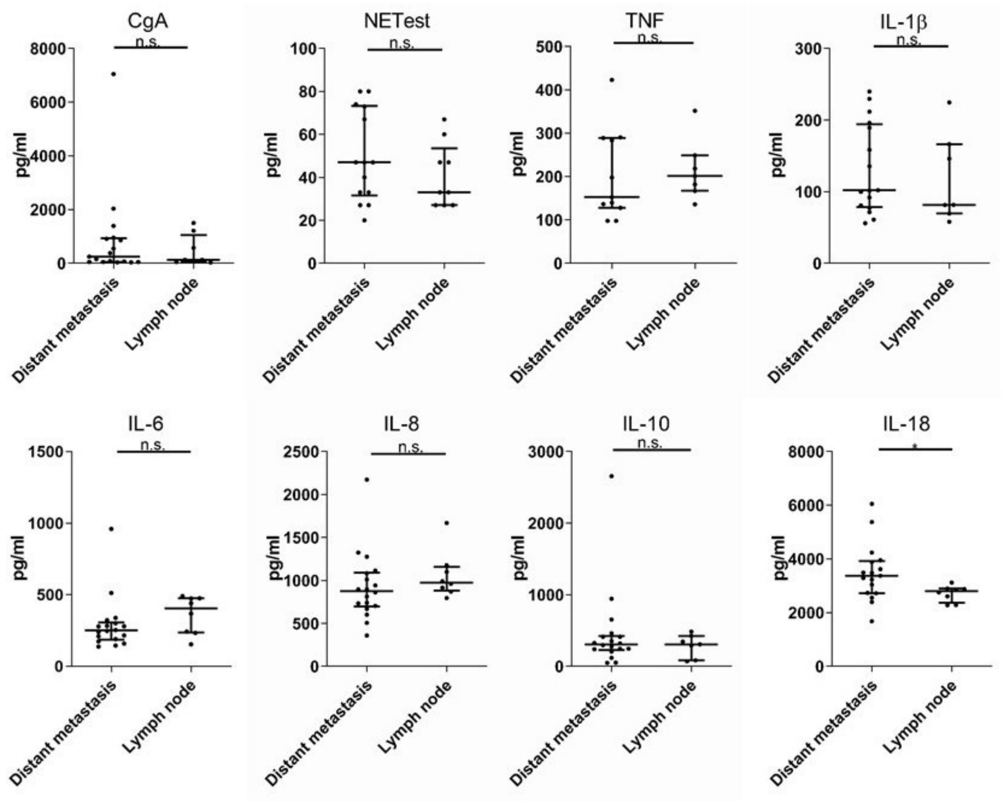

Supplementary Figure S3. **Evaluation of serum cytokines at BL according to the prevalence of metastasis.** (* p <0.05), horizontal bars indicate median and IQR)

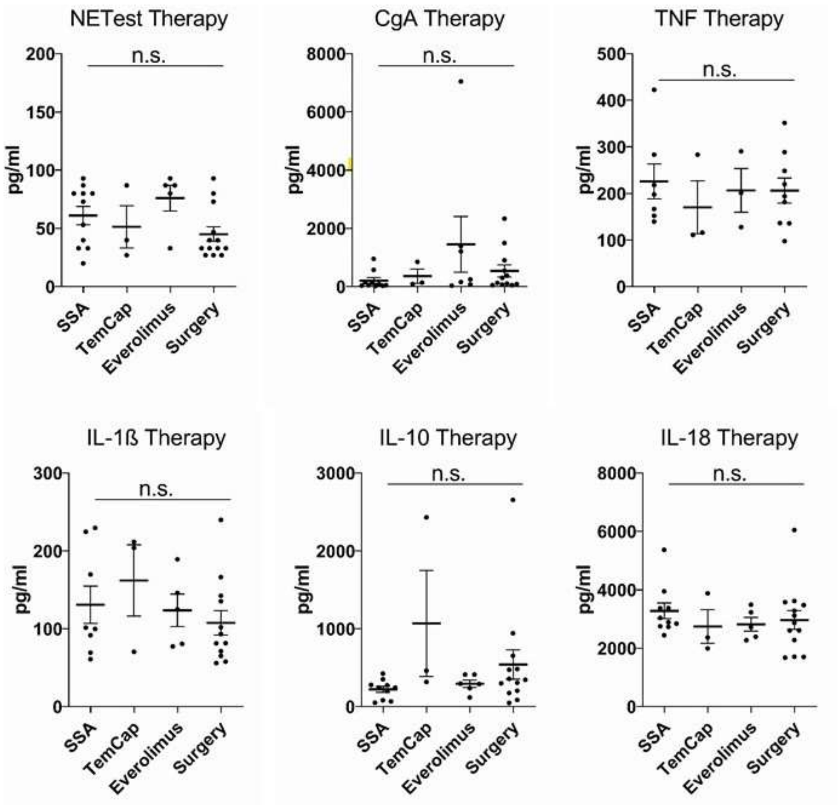

Supplementary Figure S4. **Evaluation of serum cytokines at FU in NET according therapy option.** (* p <0.05), horizontal bars indicate median and IQR)
